# *Ex vivo* comparative immunogenicity assessment (EVCIA) to determine relative immunogenicity in chronic plaque psoriasis in participants receiving Humira^®^ or undergoing repeated switches between Humira^®^ and AVT02

**DOI:** 10.1093/immadv/ltad029

**Published:** 2023-12-21

**Authors:** Kathleen Richter, Halimu N Haliduola, Jana Schockaert, Aurélie Mazy, Nataliya Reznichenko, Eric Guenzi, Fausto Berti

**Affiliations:** Alvotech Germany GmbH, Jülich, Germany; Alvotech Germany GmbH, Jülich, Germany; ImmunXperts, Gosselies, Belgium; ImmunXperts, Gosselies, Belgium; Military Hospital (Military Unit A3309) of Military-Medical Clinical Center of Southern Region, Zaporizhzhia, Ukraine; Alvotech Germany GmbH, Jülich, Germany; Alvotech Swiss AG, Zürich, Switzerland

**Keywords:** *Ex vivo* comparative immunogenicity assessment (EVCIA), AVT02, cellular immunogenicity, humoral response, adalimumab biosimilar

## Abstract

Immunogenicity against biologic medicines is ubiquitous, and it is traditionally measured by the final humoral response. However, the onset of a sustained immunogenic response begins at the cellular level with activation of T cells and maturation of naïve B cells into plasma cells. *Ex vivo* comparative immunogenicity assessment (EVCIA) of cellular immunogenicity in participants with moderate-to-severe chronic plaque psoriasis in the AVT02-GL-302 study, who received either reference product (RP) alone (non-switching arm) or switched between RP and AVT02 (switching arm) after 1:1 randomization at week 12. Peripheral blood mononuclear cells (PBMCs) were collected and cryopreserved from 28 participants at: baseline (before treatment) (week 1); pre-randomization (week 12); and week 16 and week 28 in both switching and non-switching arms. PBMCs were thawed and re-exposed to either medium alone (negative control), RP, AVT02, keyhole limpet hemocyanin (KLH) (positive control), RP+KLH, or AVT02+KLH. Samples from 10 participants (predetermined average cell viability of 75% across all timepoints) from each arm were analyzed for cytokine release after 24 hours and for Th-cell proliferation, 6 days post-seeding. Until week 28, cytokine release and Th-cell proliferation was similar at all time points in both switching and non-switching arms. Overall cellular immune response was elevated post-KLH re-exposure at all timepoints. The comparable *ex vivo* cellular immunogenicity between switching and non-switching arms complements the confirmation of interchangeability in the main study. Given the sensitivity of novel EVCIA, detecting cellular immunogenicity could be a potential outcome in predicting the immunogenicity of biologic medicines.

## Graphical Abstract

**Graphical Abstract:**
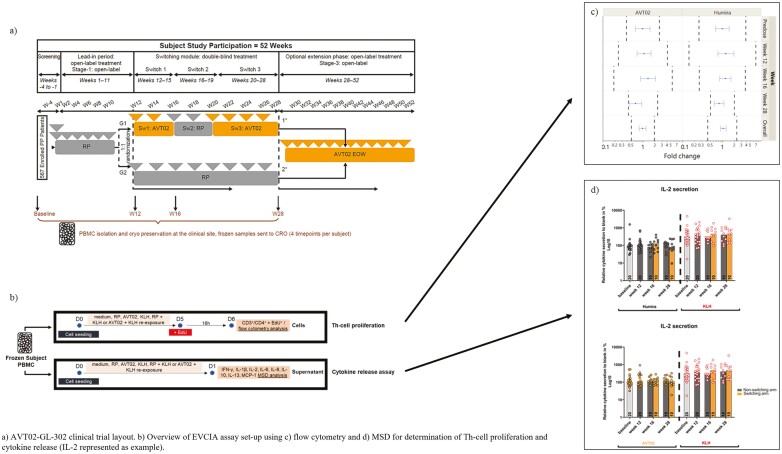

## Introduction

Plaque psoriasis is a T-cell-mediated inflammatory condition characterized by chronic erythematous plaques. Locally produced tumor necrosis factor-alpha (TNF-α) as well as resident T cells are key players in the chronic inflammation associated with psoriasis [1]. This depicts the critical role of TNF-α in the regulation of the proinflammatory cytokine cascade and local T-cell proliferation and function [2]. Anti-tumor necrosis factor (TNF) treatment has improved the quality of life of people with inflammatory diseases such as Rheumatoid arthritis (RA), Crohn’s disease, and ulcerative colitis. There are currently five US Food and Drug Administration-approved anti-TNF- biologic treatments available: infliximab (Remicade^®^), adalimumab (Humira^®^), golimumab (Simponi^®^), certolizumab pegol (Cimzia^®^), and etanercept (Enbrel^®^). Adalimumab is a recombinant human immunoglobulin 1 monoclonal antibody designed to bind and neutralize soluble and membrane-bound TNF. It specifically inhibits the interaction of p55 and p75 cell-surface TNF receptors, thereby blocking important cell-signaling pathways, such as those that lead to an inflammatory cell response [3–6].

In humans, the application of anti-TNF biologics like reference adalimumab can evoke unintended immune responses over time [7, 8]. Therefore, the evaluation of the immunogenicity of anti-TNF biologics is important to ensure the safety and therapeutic efficacy of treatments and their biosimilars. Anti-drug antibodies (ADAs) are one of the unfavorable potential outcomes of biologics, which involve both the innate and adaptive immune systems. Although the development of immunogenicity in some individuals is temporary and reflects an extent of immune tolerance, the development of high titers of ADAs over a longer period of time may considerably alter pharmacokinetics and neutralize the drug, which would likely reduce the efficacy of biologics [9].

Immunogenicity is traditionally measured by detecting ADAs in an individual’s plasma or serum during clinical trials (the humoral response). Immunogenic responses at a cellular level initiate long before the induction of ADAs, for example, T-cell activation prior to the secretion of pro- and anti-inflammatory cytokines or the development of naïve B-cell-originated plasma cells, which are the endogenous sources for ADAs [10–12]. In fact, T-cell epitopes in the primary sequences of biologics are the major inducers or harmonizers of ADA responses, depending on the type of T-cell response being stimulated (for instance, in this case, T helper or regulatory T cells, respectively) [13]. Recently, various *in vitro* methods for the evaluation of the immunogenicity of biologics have been developed, including methods to investigate T-cell proliferation or cytokine secretion from cultured peripheral blood mononuclear cells (PBMCs) [14, 15].

AVT02 is a monoclonal antibody with proposed interchangeability to reference adalimumab [2, 6, 16]. The biosimilarity of AVT02 to the reference product (RP) Humira^®^ has been previously confirmed in both a pharmacokinetic study and a confirmatory efficacy and safety study [17–20].

This paper reports *ex vivo* exploratory data from a comparative clinical study designed to support the demonstration of interchangeability between AVT02 and the RP (AVT02-GL-302; ClinicalTrials.gov Identifier: NCT04453137) [20]. The aim was to compare the cell-mediated immunogenicity in participants with moderate-to-severe chronic plaque psoriasis in the AVT02-GL-302 study who received either the RP alone or switched between the RPand AVT02 after *ex vivo* exposure of isolated PBMCs with the reference product, AVT02, or Keyhole limpet hemocyanin (KLH), using the novel *ex vivo* comparative immunogenicity assessment (EVCIA).

## Materials and methods

### Study design

The samples used in this study originated from the AVT02-GL-302 study (NCT04453137); a multicenter, randomized, double-blind, parallel-group study evaluating pharmacokinetics, efficacy, safety, and immunogenicity in participants with moderate-to-severe chronic plaque psoriasis. Participants received either the reference product or underwent repeated switches between the RP and AVT02.

**Figure 1.**
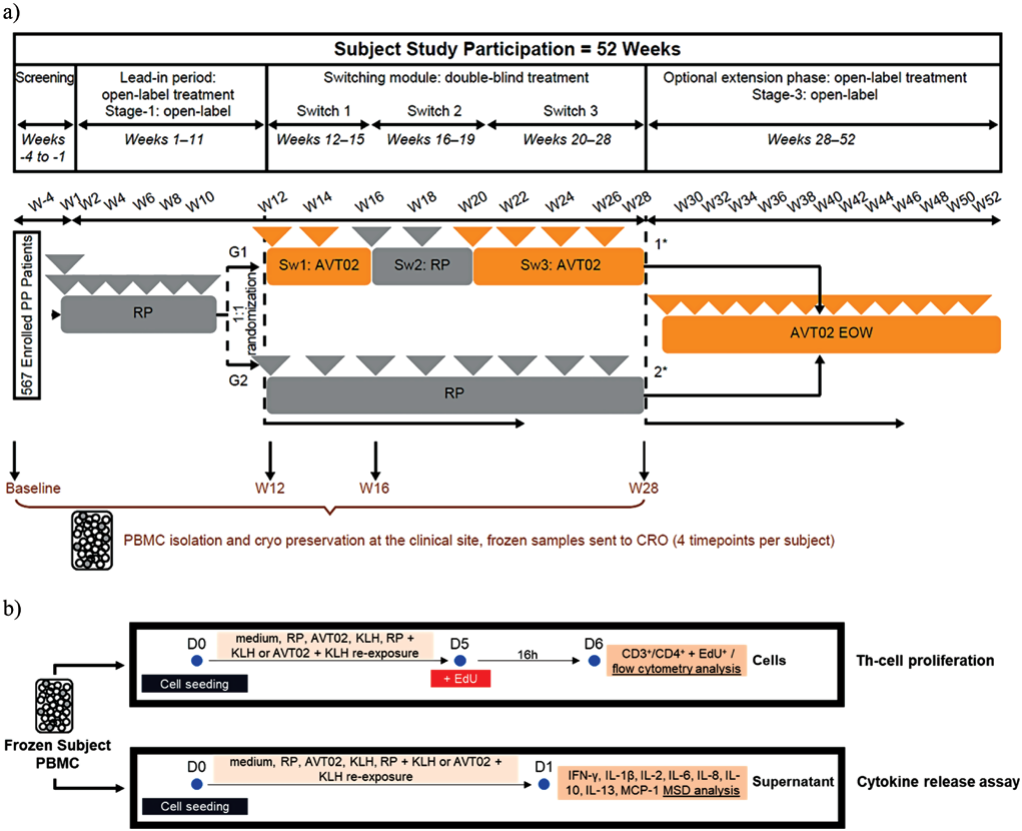
*Ex-vivo* assessment of the immunogenic potential after repeated switches between reference product and AVT02 versus reference product *in* AVT02-GL-302 interchangeability study—study schematic. (a) During the lead-in period, participants received loading dose (initial dose of 80 mg reference product [2 × 40 mg] administered subcutaneously [SC], followed by 40 mg SC given every other week (EOW) starting 1 week after the initial dose). At week 12, participants with a clinical response (psoriasis area and severity index [PASI] ≥75 [PASI75]) were randomly assigned in a 1:1 ratio to either switching arm or non-switching arm. Orange arrows indicate AVT02 administration (40 mg subcutaneous). Gray arrows indicate administration of the reference product (40 mg subcutaneous), 1* further defined as switching arm, 2* further defined as non-switching arm. For the *ex-vivo* EVCIA assessment, blood samples were drawn at the clinical site at week 1 (baseline), and before drug administration at weeks 12, 16, and 28. (b) Overview of EVCIA assay set-up using flow cytometry and MSD for determination of Th-cell proliferation and cytokine release. The cells were seeded, re-exposed to RP; AVT02; KLH (positive control); medium (negative control); RP + KLH; AVT02 + KLH for 6d (Th-cell proliferation) or 24 hours (cytokine release assay). CRO: contract research organization; PP: plaque psoriasis; Sw: switch; W: week.

The EVCIA (a Th-cell proliferation and cytokine release assay) was used to measure immunogenicity at the cellular level, and to further assess the impact of repeated switches between AVT02 and the RP. During the study, PBMC samples were isolated from whole blood sourced from the AVT02-GL-302 clinical trial and cryopreserved from 28 participants at four time points: baseline/weeks 1, 12, 16, and 28 (Fig. 1a). After study unblinding, participant samples were identified as belonging either to the non-switching or switching arm. Ten participants from each treatment arm were analyzed. They were selected based on predetermined average cell viability of 75% across all timepoints within each participant. The frozen PBMCs were thawed and re-exposed *ex vivo* to the RP, AVT02, positive- (KLH) or negative (medium) assay controls, as well as a combination of the RP (frozen, 10 μg/ml) + KLH (25 μg/ml) or AVT02 (frozen, 10 μg/ml) + KLH (25 μg/ml) for either a 6-day Th-cell proliferation assay or a 24-hour post-stimulation cytokine release assay (Fig. 1b).

### PBMC isolation

Blood samples were drawn at the clinical site (Ukraine) according to the clinical trial protocol at week 1 (baseline), and before drug administration at weeks 12, 16, and 28 (Fig. 1). Pooled blood samples (3xS-Monovette® 7.5 ml per participant, Sarstedt, cat# 11604001) were diluted 1:1 with washing buffer. PBMCs were isolated by transferring diluted blood into prefilled Leucosep tubes (Greiner, cat#227288) and spun at 400g for 35 minutes at room temperature without brakes. The PBMC layer was washed twice with washing buffer before being resuspended in 2 ml freezing medium and cryogenically stored (2 x 1 ml) until further usage.

The details of sample collection and preparation, labeling, storage, and shipment are described in the clinical trial protocol for the AVT02-GL-302 study and the respective laboratory manual.

Washing buffer was prepared using Dulbecco’s phosphate-buffered saline (Gibco, cat#14190250) and 0.5M ethylenediaminetetraacetic acid (2 mM final concentration, Gibco, cat#15575020). Freezing medium for cryogenic cells storage was prepared by adding 10% dimethyl sulfoxide (DMSO, AppliChem GmbH, cat#A3672,0250) to 90% fetal bovine serum (Gibco, cat#16140071). KLH was purchased from Enzo (cat# ALX-202-064).

### *Ex vivo* EVCIA

To evaluate the cell-mediated immunogenicity of the RP and AVT02, a novel EVCIA was developed and performed using freshly isolated and subsequently cryopreserved PBMCs from study participants, which included both human innate and adaptive immune cells.

For both, the Th-cell proliferation assay and the cytokine release assay, PBMCs were seeded using tissue culture-treated 96-well round-bottom microplates at 2.0 × 10^5^ cells per well separately for each assay. For both the assays, the following test conditions were added to defined wells: the RP, AVT02, positive- (KLH) or negative (medium) assay controls, as well as a combination of the RP (frozen, 10 μg/ml) + KLH (25 μg/ml) or AVT02 (frozen, 10 μg/ml) + KLH (25 μg/ml). The assays were each performed in three replicate wells per test condition. Details are given below.

#### Th-cell proliferation assay

PBMC cultures were pulsed on Day 5 with 5-ethynyl-2ʹ-deoxyuridine (EdU) for approximately 16 hours. On Day 6, cluster of differentiation (CD) 4^+^ Th-cell proliferation was assessed by measuring EdU incorporation via flow cytometry. Cells were fluorescently stained for live/dead differentiation and Th-cell surface markers (CD3 and CD4); the cells were then fixed, permeabilized, and the incorporated EdU was stained with a fluorescent azide. All flow cytometry data were acquired with a MACS Quant 10 (Miltenyi) and analyzed using FlowLogic software. Proliferating Th cells were defined as CD3^+^CD4^+^EdU^+^ cells and the gating strategy applied to identify them is described below.

A lymphocyte gate was applied on a forward scatter (FSC)/side scatter (SSC) dot plot to separate the debris from cells. Doublets were excluded using a double exclusion (FSC-A/FSC-H and SSC-A/SSC-H). The live cells were then gated using a viability dye. The T cells were gated based on their CD3 expression, which was followed by CD4 expression. Finally, proliferating CD3^+^CD4^+^cells were gated on a CD4-EdU dot plot, yielding CD3^+^CD4^+^EdU^+^ cells.

The statistical analyses were performed with the statistical software SAS JMP version 15 (SAS Institute Inc., Cary, NC, 1989–2021). T-cell stimulation index (SI) per donor and test condition (see Section Ex vivo EVCIA. for test conditions), as measured by 3H-thymidine incorporation, was calculated by dividing the geometric mean of EdU^+^ counts of the test condition by the geometric mean of EdU^+^ counts of the negative control (medium only) condition. Statistical analysis was performed on log-transformed SI values, as the original data follow log-normal distribution. An equivalence test was performed to evaluate if switching arm participants responded similarly to each stimulation compared with the non-switching arm participants [21]. The cut-off for a non-relevant effect was determined based on the variation in log(SI) data from the non-switching arm. The standard deviation was estimated as the residual variation of a model on data from the non-switching arm only, per stimulation. An intercept only (no predictors) model was fitted per time point. A model was fitted with participants as a random effect, and time as a fixed effect for the overall comparison across all time points. A relevant difference was then specified as 2.5× the residual variation from these models. Similar models were fitted on data including non-switching arm and switching arm participants, with treatment arm added as a fixed effect (including an interaction with time for the overall model). A type III estimate for the treatment arm was calculated with 90% confidence intervals (CIs). Equivalence was concluded if these CIs did not exceed a relevant difference. Results were back-transformed by exponentiating, and all differences and equivalence limits could be interpreted as fold changes of the test (switching) arm versus the reference (non-switching) arm.

#### Cytokine release assay

Twenty-four hours after the PBMCs had been added to the culture, the tissue culture-treated 96-well round-bottom microplates were centrifugated at 300g for 10 minutes at room temperature, and the supernatant was harvested and transferred into a new 96-well non-sterile V-bottom well plate. After a second centrifugation at 800g for 5 minutes at room temperature, the supernatant was harvested and transferred into a new 96-well non-sterile V-bottom well plate and stored at –80°C until samples were shipped to Alvotech Germany GmbH for the final analysis, where samples were stored at ≤ –65°C until analysis.

The simultaneous quantitation of interferon gamma (IFN-γ), interleukin-1 beta (IL-1β), interleukin-2 (IL-2), interleukin-6 (IL-6), interleukin-10 (IL-10), interleukin-13 (IL-13), and monocyte chemoattractant protein-1 (MCP-1) were performed with a sandwich assay on separate 96-well multiplex microtiter plates. IL-8 cytokine measurements were executed by a single plex assay using the electrochemiluminescence multiplex assay (Meso Scale Discovery platform) technology according to the manufacturer’s instructions.

Supernatant samples were thawed and centrifuged for 1 minute at 1300 rpm followed by 6- (IFN-γ, IL-1β, IL-2, IL-6, IL-10, IL-13, and MCP-1) and 10-fold (IL-8) dilutions, respectively, for quantifying cytokine profiles.

Data analyses were performed using Meso Scale DISCOVERY WORKBENCH software to create a calibration curve for each analyte using a 4-parameter fit (1/*Y*^2^ weighting function) as well as to calculate the final concentration of the respective analyte in the sample (in reference to the standard curve of each analyte). Final analysis and data representation are shown as relative percentage secretion to the relevant negative control sample (medium only), with the control sample set to 100%.

Statistical analysis for the cytokine release assay was performed using GraphPad Prism 9 using the following tests based on suitability: Kruskal–Wallis—multiple comparison—nonparametric, multiple Mann–Whitney test, two-way analysis of variance (mixed model), or multiple *t*-test—unpaired.

## Results

### Th-cell proliferation assay

**Figure 2.**
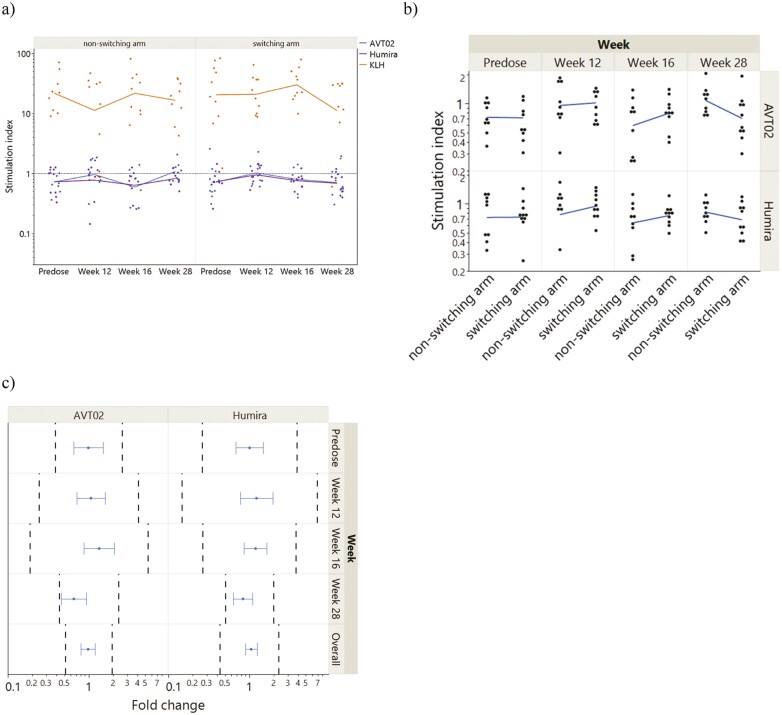
Equivalence test for T-cell proliferation comparing response after the reference product, AVT02, or KLH stimulation at different timepoints in switching arm versus non-switching arm participants. PBMCs were stimulated for 5 days in the presence of reference product (RP) Humira, AVT02, or KLH and pulsed on Day 5 with 5-ethynyl-2ʹ-deoxyuridine (EdU) for approximately 16 hours. The proliferation of T cells was determined via flow cytometry by calculating the number of cells within the CD4+/ EdU+ gate. T-cell stimulation index (SI) per donor and test was calculated by dividing the geometric mean of EdU+ counts (within the CD4+ T-cell population) of the test condition by the geometric mean of EdU+ counts of the negative control (medium only) condition. (a) SI values are divided into participants from the switching arm (right) and participants from the non-switching arm (left). (b) SI per stimulation and time point for each treatment arm. Each dot represents the SI of each participant, and the lines connect the geometric mean across all participants per treatment arm. (c) Equivalence test of switching arm compared with non-switching arm after re-exposure to AVT02 and the reference product. Dashed black reference lines represent the equivalence limits based on the variation in outcome of non-switching arm participants. Blue dots represent the fold change between switching arm and non-switching arm participants, with error bars representing 90% CIs: confidence interval; KLH: keyhole limpet hemocyanin; SI: stimulation index.

One-hundred and twelve cryopreserved PBMC samples were collected for this study from a total of 20 participants at the four different time points: baseline, weeks 12, 16, and 28. All participant samples showed an elevated response to KLH re-exposure compared to negative control, as assessed by CD3^+^CD4^+^EdU^+^ cells (data available upon request). No significant difference in Th-cell proliferation was observed after the RP and AVT02 alone re-exposure in the non-switching arm compared with the switching arm at all time points, except at week 28 where SIs were higher in the non-switching arm compared with the switching arm. Compared with negative controls, the geometric mean SIs for each stimulation at baseline, weeks 12, 16, and 28, for the participants after re-exposure to the RP or AVT02 were between 0.66 and 0.85, and for KLH between 14 and 24.3 (Fig. 2a).

#### Equivalence test

An overview of the equivalence limits, average fold changes, and 90% CI is shown in Supplementary Table S1. The geometric mean SIs per participant, time, and stimulation condition for all participants are shown in Fig. 2b. It shows that geometric mean SIs and distribution of SI of each participant after re-exposure to AVT02 and RP were similar between the switching arm and non-switching arms. Fig. 2c shows the results of the equivalence test described in Section Th-cell proliferation assay. The 90% CI for the difference between switching arm and non-switching arm participants after re-exposure to AVT02 and RP were within the equivalence margins. Except where there is a significant difference or variation in response to any stimulation condition at any time point between switching and non-switching arms.

### Cytokine release assay

**Figure 3.**
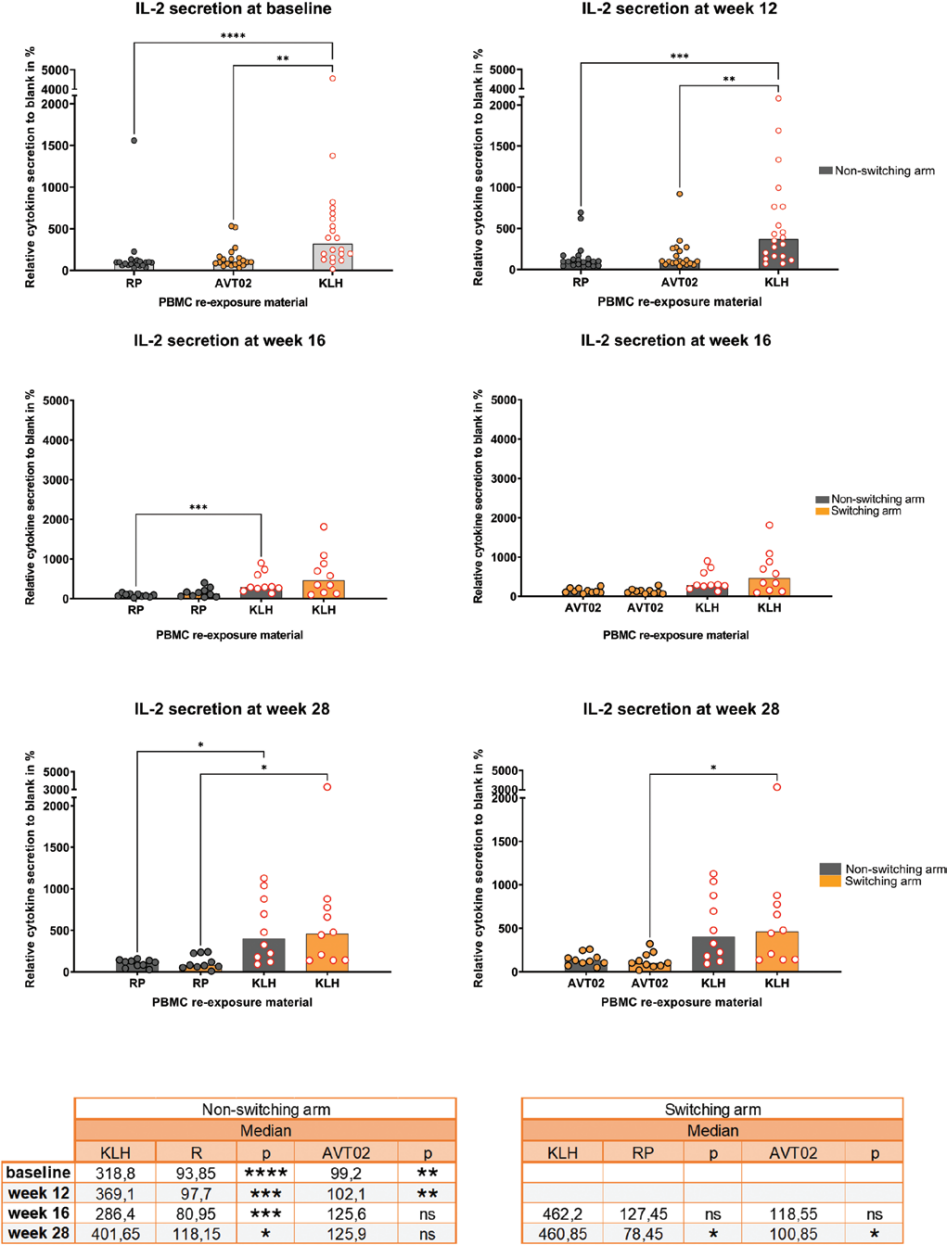
Relative cytokine secretion after *ex vivo* stimulation of PBMCs with the reference product, AVT02, or KLH at different time points. IL-2 release, after reference product Humira or AVT02 *ex vivo* PBMC stimulation of subject samples collected from the AVT02-GL-302 clinical study at different timepoints, is represented as relative secretion to negative control in %, it was set to 100%. Blood samples were obtained from healthy subjects at different time points (Fig. 1—study design), PBMC’s were isolated and frozen at site, stored before sending it to the CRO (ImmunXpert). At the CRO site, cells were thawed and re-exposed to reference product Humira, AVT02, KLH, reference product Humira + KLH or AVT02 + KLH for 24 hours and supernatant was collected, frozen, and shipped to the Alvotech Germany GmbH site. Multiplex MSD assays were performed to determine IL-2 secretion as a relative secretion with respect to the negative control (medium). Data points represent the average of two technical replicates per sample, median of all data points per treatment is represented as a bar and as an overview of relative values in tabular form below. Subjects defined to the non-switching arm (dark gray bar) or switching arm (orange bar) with *n* = 20 at predose and week 12, divided in *n* = 10 per treatment arm after week 12 (Fig. 1—study design). Re-exposure samples are represented as: dark gray dots for reference product Humira re-exposure, orange dots with gray border for AVT02 re-exposure, white dots with red border for KLH re-exposure. Kruskal–Wallis test—multiple comparison: *P* value of values: * ≤ 0.05, ** ≤ 0.01, *** <0.001, **** <0.0001, ns = not significant calculated using all data points per time point. IL: interleukin; KLH: keyhole limpet hemocyanin; PBMC: peripheral blood mononuclear cell.

Overall, increased secretion of IFN-γ, IL-2, and IL-6 (Fig. 3 and Supplementary Fig. S1 and IL-1β, IL-8, IL-10, IL-13, and MCP-1 (Supplementary Fig. S1) was detected after re-exposure with KLH. While no significant differences were seen between treatment arms after re-exposure to the RP or AVT02 at all time points.

Overall, the cytokine elevation was similar compared to the negative control. Although, IL-6 elevation was relatively marked compared to IL-2 and IFN-γ. The augmentation in IL-6 secretion after KLH re-exposure showed the highest fold change compared with the RP (100-fold) or AVT02 (31-fold) in the baseline samples; all other cytokines showed fold changes between 3.2–10.47 (RP) and 1.2–5.5 (AVT02) (data available upon request). IL-6 is a significant biomarker for T-cell or secondarily B-cell response to a foreign protein.

#### EVCIA analysis of pro-inflammatory cytokines: IL-2, IFN-γ, and IL-6

All subject samples demonstrated an increase in IL-2 cytokine secretion by PBMCs *ex vivo* after re-exposure to KLH, compared with the negative control (medium only—unstimulated condition) (Fig. 3a) at all time points. However, the RP and AVT02 following re-exposure showed only marginal increase in IL-2 secretion at all time points, compared with negative control.

At baseline as well as week 12, the IL-2 secretion was significantly higher after KLH re-exposure compared to both the RP and AVT02 re-exposure. At week 16, in the switching arm, no significant difference was observed in IL-2 secretion after the RP versus KLH re-exposure, while in the non-switching arm the difference between the two was significant. Although, at week 16, no differences were observed in IL-2 secretion after AVT02 versus KLH re-exposure in both the arms. At week 28, the difference in IL-2 secretion was remained significant after the RP versus KLH re-exposure in both the arms, no difference was observed after AVT02 versus KLH re-exposure in the non-switching arm, a difference was observed after AVT02 versus KLH re-exposure in the switching arm.

**Figure 4.**
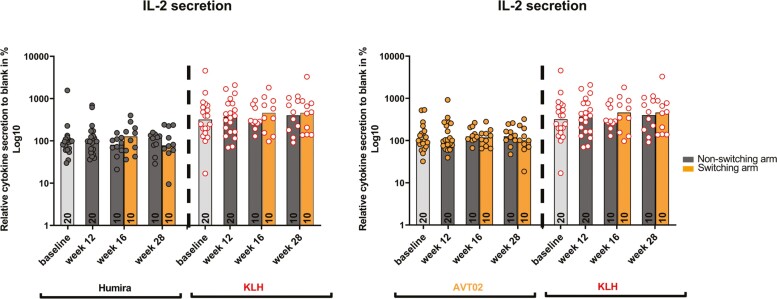
Comparison of IL-2 secretion between non-switching and switching arms in participant samples collected at different time points after reference product, AVT02, or KLH *ex vivo* PBMC stimulation. IL-2 secretion is represented as relative secretion to negative control in %, negative control was set to 100%. Multiplex MSD assays were performed to determine IL-2 secretion as a relative secretion with respect to the negative control (medium). Data points represent the average of two technical replicates per sample, median of all data points per treatment is represented as a bar. Participants in the non-switching arm (dark gray bar, *n* = 10 per treatment arm after week 12) or switching arm (orange bar, *n* = 10 per treatment arm after week 12). Values of samples are represented as: dark gray dots for reference product re-exposure, orange dots with gray border for AVT02 re-exposure and white dots with red border for KLH re-exposure. Kruskal–Wallis test—multiple comparison showed no significance between timepoints within each defined treatment. IL: interleukin; KLH: keyhole limpet hemocyanin; MSD: meso scale discovery; PBMC: peripheral blood mononuclear cell.

Overall, IL-2 stimulation following KLH re-exposure appeared to be similar between baseline and week 12, with a similar pattern in the non-switching arm at weeks 16 and 28. In the switching arm, the KLH re-exposure response was slightly elevated at week 16 and week 28, reaching to a similar level at week 28 in the non-switching arm. Kruskal–Wallis test showed no significant difference in IL-2 secretion between the treatment arms, at all timepoints after re-exposure to the RP or AVT02 (Fig. 4).

Similarly, all samples demonstrated an increase in IFN-γ cytokine secretion after re-exposure to KLH compared with the negative control (medium only—unstimulated condition) at all time points (Supplementary Fig. S1). Although only a marginal increase in cytokine secretion was observed in IFN-γ secretion after both the RP and AVT02 re-exposure compared with negative control at all time points.

At baseline and week 12, the difference in IFN-γ secretion was significantly higher after KLH re-exposure compared to both the RP and AVT02 re-exposure.

At weeks 16 and 28, AVT02 re-exposure led to a stronger IFN-γ cytokine secretion response with no significant difference between AVT02 versus KLH re-exposure in both the arms. Although, both at weeks 16 and 28 in both the arms, the significant difference was observed after the RP versus KLH re-exposure.

Overall, IFN-γ stimulation after the KLH response appears to be higher at week 12 than at baseline. In the switching arm, at both weeks 16 and 28 it was similar to week 12. While, in the non-switching arm, at week 16 the response was similar to baseline and at week 28 was similar to week 12.

Unlike with other cytokines, all samples demonstrated an highly increase in IL-6 cytokine secretion following KLH re-exposure compared with the negative control (medium only—unstimulated condition) at all time points (Supplementary Fig. S1). Although, as like other cytokines only a marginal increase in IL-6 secretion was observed following both the RP and AVT02 re-exposure compared with the negative control.

At baseline and week 12, differences were observed in IL-6 secretion after both the RP and AVT02 re-exposure versus KLH re-exposure. Similarly, at weeks 16 and 28 no differences were observed after AVT02 versus KLH re-exposure in the switching arm, however, in the non-switching arm the differences were significant. It suggests that AVT02 re-exposure led to a stronger response at baseline and week when compared with the RP. At weeks 16 and 28, significant differences were observed in both arms after the RP versus KLH re-exposure.

Overall, after KLH re-exposure, the IL-6 stimulation appears to be higher at baseline compared with week 12. At week 16, it was similar to baseline in both the arms. At week 28, stimulation after KLH re-exposure in the switching arm showed a lower IL-6 response compared with the non-switching arm and was also reduced compared to week 16. In the non-switching arm, the response was elevated at week 28 compared with baseline and week 16.

**Figure 5.**
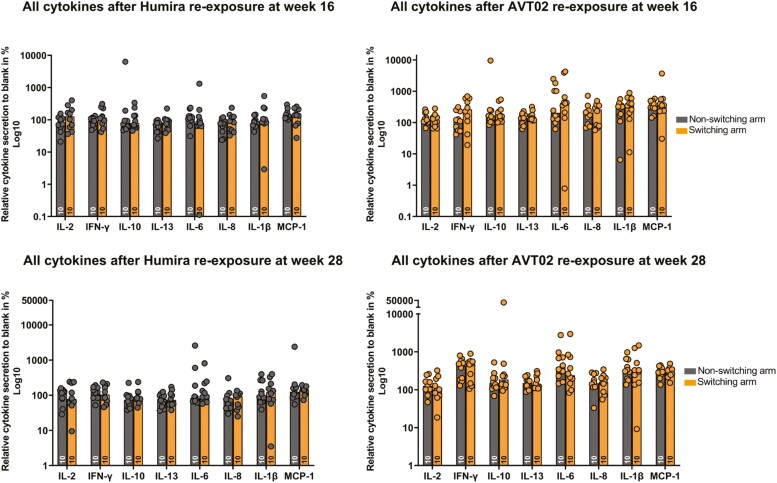
EVCIA biomarker profile comparison at weeks 16 and 28 following stimulation with the reference product or AVT02 in the switching arm versus non-switching arm. Re-exposure samples are represented as: dark gray dots for reference product re-exposure and orange dots with gray border for AVT02 re-exposure. The unpaired *t* test showed no significant difference in both timepoints, between both arms and for each cytokine. *EVCIA ex vivo* comparative immunogenicity assessment, IFN-γ: interferon gamma; IL: interleukin; KLH: keyhole limpet hemocyanin; MCP-1: monocyte chemoattractant protein-1.

A comparison for all EVCIA biomarkers at weeks 16 and 28 in the non-switching and switching arms is shown in Fig. 5. Statistical analyses via unpaired *t* test showed no significant difference between both the arms at both weeks 16 and 28 for each cytokine (Fig. 5). For baseline and week 12, the data are shown in Supplementary Fig. S2. There were no immediate critical cell-mediated immune response, particularly for T-cell-related IL-2 or IFN-γ. In addition, no significant differences were observed in the response at weeks 16 and 28 comparing the non-switching arm with the switching arm after re-exposure.

#### EVCIA analysis: all cytokines

Overall, cytokine secretion after re-exposure with KLH was higher compared to the negative control and similar in both treatment arms over time, statistical analysis via Kruskal–Wallis test showed no significant difference between the treatment arms for each cytokine (Supplementary Fig. S1**).** Increased concentration was more pronounced for IL-8 followed by IL-6 compared to all other cytokines, which were similarly elevated compared to the negative control (see Section Cytokine release assay). For all re-exposure conditions and in both arms, cytokine secretion did not differ significantly between baseline and week 28. Overall, a slightly higher sample distribution was observed after KLH re-exposure. Relative cytokine secretion at each time point after the RP, AVT02, or KLH re-exposure for IL-1β, IL-8, IL-10, IL-13, and MCP-1 are shown in Supplementary Fig. S3.

## Discussion

The goal of this exploratory analysis was to evaluate the *ex vivo* immunogenic potential of a biosimilar AVT02 compared with the RP (Humira^®^), using PBMCs purified from participant samples treated with either the RP alone or with repeated switches between the RP and AVT02 in the AVT02-GL-302 clinical study. We used the novel EVCIA assay to measure the immunogenicity *ex vivo* at the cellular level, which consists of the Th-cell proliferation assay and the cytokine release assay. The findings of this study are in line with the AVT02-GL-302 clinical study which supports demonstration of the potential interchangeability of the RP and AVT02 as anti-TNF treatments for individuals with inflammatory diseases, such as chronic plaque psoriasis.

The relative Th-cell proliferation was lower (SI < 1) after the RP and AVT02 re-exposure, whereas KLH re-exposure showed strongly positive responses (SI between 14 and 24.3), as expected. Despite the fact that there was no pronounced time-response trend after KLH stimulation in either arm, except at week 16, when a peak in SI value was observed in both treatment arms, the response to each stimulation in the switching arm samples is not greater than the non-switching arm.

The final concentration of IFN-γ, IL-1β, IL-2, IL-6, IL-8, IL-10, IL-13, and MCP-1 in the human PBMC supernatant samples varied depending on stimulation type (negative control: medium, the RP, AVT02 or positive control: KLH). An increased secretion of all tested biomarkers was observed after re-exposure with the positive control (KLH). The highest increase was detected for the pro-inflammatory cytokines: IL-8 (one of the major mediators of the inflammatory response, ~ 23 000–25 000 pg/ml), followed by IL-6 (~ 2100–2500 pg/ml), and the pro-inflammatory chemokine MCP-1 (~ 1000–1300 pg/ml) (data available upon request). The highest signal detection of these specific biomarkers is equivalent to a similar study [8] and shows that the cytokine release assay is functioning on the chosen stimuli and has sufficient sensitivity to detect a cell response.

There was no significant increase among pro-inflammatory Th cell-specific cytokines like IL-2 (lowest detected concentration among all tested biomarkers) or IFN-γ, as well as the anti-inflammatory T-regulatory cell-specific IL-10 or Th2 IL-13 cytokines compared to IL-8 or IL-6 following KLH stimulation. The increased secretion of IL-8 or IL-6 may have been due to the differential activation of different cell types within the PBMC population, such as monocytes, natural killer cells, or B cells, by KLH compared with IL-2, which is uniquely delimited to the activation of Th cells [22]. The cell response generally relies on cells representing the innate immunity (macrophages, monocytes, and dendritic cells), rather than cells of the adaptive immunity (T cells and B cells). No elevated cytokine expression was observed after the RP or AVT02 re-exposure. This shows that innate immunity cell populations are not falsely recognizing both drugs, such as by toll-like receptors, which is another sensitive mechanism involved in the onset of immunogenicity [23]. In addition, there was no preferable Th cell-subtype activation, as evidenced by the lack of elevation in IFN-γ secretion (a Th1-defined biomarker) and IL-13 (a Th2-defined biomarker) after the RP or AVT02 re-exposure compared with the negative control.

There was no elevation in the cytokine secretion after the RP or AVT02 re-exposure across all time points compared to KLH re-exposure, supporting the assumption that the cytokine release syndrome is highly unlikely with the RP or AVT02, as confirmed during the AVT02-GL-302 clinical study [24]. Moreover, it recommends the inclusion of the cytokine secretion analysis at a later stage in the study, approximately 6–7 days post-exposure. In addition, the biomarker read-out over the study time shows that the cell profile during switching between the RP and AVT02, the priming of the immune cells, and the subsequent specific humoral response after re-exposure to the RP or AVT02 were similar and consistent with the non-switching arm.

Given the fact that the RP can inhibit the pro-inflammatory cytokines IL-2, INF- γ, or IL-6 [25, 26], this study re-exposed PBMCs to a combination of the RP + KLH or AVT02 + KLH. Following 24-hour exposure, data showed no significant inhibition of these pro-inflammatory cytokines secretion in comparison with KLH stimulation alone (Supplementary Fig. S4), the exposure time was short. Hence, an additional B-cell-specific assay evaluating immunogenicity could support the detection of fluctuations in antibody production (such as ADAs) as a primary immune response.

High sample distribution was observed during the study, particularly after KLH re-exposure. However, responses were generally higher than the median across all time points. A slightly higher distribution can result from non-depletion of the PBMC population of CD8^+^ cells to reduce interference, and to measure the CD4^+^ T-effector response more precisely. A wider distribution within one population is not unusual, participant-related factors that also need to be considered such as an unknown disease state, the presence of pre-existing antibodies against the therapeutic protein, and the genetic background that contributes to the immunogenicity of therapeutic proteins [27, 28].

The novel EVCIA assay is sensitive enough to detect a cellular response after KLH re-exposure and no stimulation of immune cells, triggered by the RP or AVT02, can be detected within the limit of this experimental approach. The absence of an immediately critical cell-mediated immune response, as well as similar responses at weeks 16 and 28, between the non-switching and the switching arms after the RP or AVT02 re-exposure, support a similar receptor specific recognition of both the RP and AVT02. All considered, detecting small biomarker secretion differences in the pg/ml range between the negative control (medium) and RP or AVT02 re-exposure across all time points (data not shown) shows the sensitivity of this assay.

The findings from this study need to be considered within the context of the study’s limitations. First, this study required the blood samples to be treated rapidly after collection, due to the sensitivity of the EVCIA. Small delays during the blood sample collection and handling process could have an impact on the overall cell response and subsequent data, such as cell viability. Secondly, due to the relatively small sample sizes and the exploratory nature of the analyses, the identification of potential differences or similarities between the properties of AVT02 and RP Humira^®^ need further confirmation in prospectively designed, randomized, controlled clinical studies.

## Conclusion

This study found that the *ex-vivo* cellular immune response from participants with moderate-to-severe chronic plaque psoriasis was highly similar between those who received the RP exclusively versus those who switched treatments between the RP and AVT02, over a 28-week study period. This is consistent with the results from the main study, which support a demonstration of interchangeability. Data were generated using the novel EVCIA, which provides a unique evaluation of both qualitative and quantitative changes in the cellular immunogenic response induced by switching conditions *in vivo*. Initial cellular response could be a valuable additional outcome to predict overall immune response to biologic medicines, currently typically measured by the later development of ADAs and Nabs.

## Supplementary Material

ltad029_suppl_Supplementary_Tables_S1_Figures_S1-S4

## Data Availability

The datasets generated and/or analyzed during the current study are available from the sponsor on reasonable request.

## Abbreviations

ADAs: anti-drug antibodies
CD: cluster of differentiation
CRO: contract research organization
EdU: Edu 5-ethynyl-2’-deoxyuridine
EOW: every other week
FSC: forward scatter
EVCIA: *ex vivo* comparative immunogenicity assessment
IFN-γ: interferon gamma
IL: interleukin
IL-10: interleukin-10
IL-13: interleukin-13
IL-1β: interleukin-1 beta
IL-2: interleukin-2
IL-6: interleukin-6
KLH: keyhole limpet hemocyanin
MCP-1: monocyte chemoattractant protein-1
PBMC: peripheral blood mononuclear cells
PP: plaque psoriasis
RA: rheumatoid arthritis
RP: reference product
SI: stimulation index
SSC: side scatter
Sw: switch
TNF-α: tumor necrosis factor-alpha
W: week

## Trial Registration

This main study is registered with www.clinicaltrials.gov, NCT04453137.

## References

[1] Schön MP , Boehncke WH. Psoriasis. N Engl J Med 2005; 352(18):1899–912. 10.1056/NEJMra041320 15872205

[2] Herrier RN. Advances in the treatment of moderate-to-severe plaque psoriasis. Am J Health Syst Pharm 2011; 68(9):795–806. 10.2146/ajhp100227 21515863

[3] EMA. *Humira (adalimumab). Summary of Product Characteristics*. 2021. https://www.ema.europa.eu/en/documents/overview/Humira-epar-medicine-overview_en.pdf (16 March 2023, date last accessed).

[4] FDA. Humira (adalimumab) US Package Insert. AbbVie, Inc., 2021. https://www.accessdata.fda.gov/drugsatfda_docs/label/2014/125057s356lbl.pdf (16 March 2023, date last accessed).

[5] Tran BN , Chan SL, Ng C et al. Higher order structures of adalimumab, infliximab and their complexes with TNFα revealed by electron microscopy. Protein Sci 2017; 26(12):2392–8. 10.1002/pro.3306 28940886 PMC5699491

[6] Atiqi S , Hooijberg F, Loeff FC et al. Immunogenicity of TNF-inhibitors. Front Immunol 2020; 11:312. 10.3389/fimmu.2020.00312 32174918 PMC7055461

[7] Strand V , Goncalves J, Isaacs JD. Immunogenicity of biologic agents in rheumatology. Nat Rev Rheumatol 2021; 17(2):81–97. 10.1038/s41584-020-00540-8 33318665

[8] Joubert MK , Deshpande M, Yang J et al. Use of in vitro assays to assess immunogenicity risk of antibody-based biotherapeutics. PLoS One 2016; 11(8):e0159328. 10.1371/journal.pone.0159328 27494246 PMC4975389

[9] Garcês S , Demengeot J. The immunogenicity of biologic therapies. Curr Probl Dermatol 2018; 53:37–48. 10.1159/000478077 29131036

[10] Karle AC. Applying MAPPs assays to assess drug immunogenicity. Front Immunol 2020; 11:698. 10.3389/fimmu.2020.00698 32373128 PMC7186346

[11] Cohen S , Chung S. In vitro immunogenicity prediction: bridging between innate and adaptive immunity. Bioanalysis 2021; 13(13):1071–81. 10.4155/bio-2021-0077 34124935

[12] Harding FA , Stickler MM, Razo J et al. The immunogenicity of humanized and fully human antibodies: residual immunogenicity resides in the CDR regions. Mabs 2010; 2(3):256–65. 10.4161/mabs.2.3.11641 20400861 PMC2881252

[13] Jawa V , Terry F, Gokemeijer J et al. T-cell dependent immunogenicity of protein therapeutics pre-clinical assessment and mitigation-updated consensus and review 2020. Front Immunol 2020; 11:1301. 10.3389/fimmu.2020.01301 32695107 PMC7338774

[14] Duke BR , Mitra-Kaushik S. Current in vitro assays for prediction of T cell mediated immunogenicity of biotherapeutics and manufacturing impurities. J Pharm Innov 2020; 15:202–18. 10.1007/s12247-019-09412-5

[15] Goodell V , dela Rosa C, Slota M et al. Sensitivity and specificity of tritiated thymidine incorporation and ELISPOT assays in identifying antigen specific T cell immune responses. BMC Immunol 2007; 8:21. 10.1186/1471-2172-8-21 17850666 PMC2034595

[16] Humira (adalimumab) Summary of Product Characteristics. European Medicines Agency (EMA), 2021. https://www.ema.europa.eu/en/documents/product-information/humira-epar-product-information_en.pdf (5 October 2022, date last accessed).

[17] Hukyndra (adalimumab) Summary of Product Characteristics. European Medicines Agency (EMA), 2022. https://www.ema.europa.eu/en/documents/product-information/hukyndra-epar-product-information_en.pdf (5 October 2022, date last accessed).

[18] Feldman SR , Reznichenko N, Pulka G et al. Efficacy, safety and immunogenicity of AVT02 versus originator adalimumab in subjects with moderate to severe chronic plaque psoriasis: a multicentre, double-blind, randomised, parallel group, active control, phase III study. BioDrugs 2021; 35(6):735–48. 10.1007/s40259-021-00502-w 34657274 PMC8520467

[19] Richter O , O’Reilly T, Guerrieri D et al. GP2017-HCF, a high concentration formulation, demonstrates similar pharmacokinetics, immunogenicity and safety to GP2017, an approved adalimumab biosimilar. Expert Opin Biol Ther 2023; 23(8):749–58. 36039657 10.1080/14712598.2022.2117546

[20] Feldman S. 0139: a clinical study designed to support a demonstration of interchangeability between AVT02 and reference adalimumab (Humira®). In: Session: (0123–0149) Miscellaneous Rheumatic and Inflammatory Diseases Poster I. American College of Rheumatology (ACR), 2022. https://www.eventscribe.net/2022/ACRConvergence/fsPopup.asp?Mode=presInfo&PresentationID=1128607 (16 March 2023, date last accessed).

[21] Tsong Y , Xiaoyu D, Meiyu S. Development of statistical methods for analytical similarity assessment. J Biopharm Stat 2017; 27(2):197–205. 27977326 10.1080/10543406.2016.1272606

[22] Sojka DK , Bruniquel D, Schwartz RH et al. IL-2 secretion by CD4+ T cells in vivo is rapid, transient, and influenced by TCR-specific competition. J Immunol 2004; 172(10):6136–43. 10.4049/jimmunol.172.10.6136 15128800

[23] Moussa EM , Kotarek J, Blum JS et al. Physical characterization and innate immunogenicity of aggregated intravenous immunoglobulin (IGIV) in an in vitro cell-based model. Pharm Res 2016; 33(7):1736–51. 10.1007/s11095-016-1914-4 27037576

[24] Vessillier S , Eastwood D, Fox B et al. Cytokine release assays for the prediction of therapeutic mAb safety in first-in man trials—whole blood cytokine release assays are poorly predictive for TGN1412 cytokine storm. J Immunol Methods 2015; 424:43–52. 10.1016/j.jim.2015.04.020 25960173 PMC4768082

[25] Shao Y , Cheng Z, Li X et al. Immunosuppressive/anti-inflammatory cytokines directly and indirectly inhibit endothelial dysfunction—a novel mechanism for maintaining vascular function. J Hematol Oncol 2014; 7:80. 10.1186/s13045-014-0080-6 25387998 PMC4236671

[26] Walscheid K , Weinhage T, Foell D et al. Effect of adalimumab on peripheral blood mononuclear cells in non-infectious uveitis. Ocul Immunol Inflamm 2019; 27(2):330–7. 10.1080/09273948.2017.1374415 29020495

[27] Schellekens H. Bioequivalence and the immunogenicity of biopharmaceuticals. Nat Rev Drug Discov 2002; 1(6):457–62. 10.1038/nrd818 12119747

[28] Schellekens H. Factors influencing the immunogenicity of therapeutic proteins. Nephrol Dial Transplant 2005; 20(6):vi3–9. 10.1093/ndt/gfh1092 15958824

