# Supplementary material for: Ex vivo comparative immunogenicity assessment (EVCIA) to determine relative immunogenicity in chronic plaque psoriasis in participants receiving Humira® or undergoing repeated switches between Humira® and AVT02
Source: Immunother Adv. 2023 Dec 21;4(1):ltad029. doi: 10.1093/immadv/ltad029 (PMC10808003; doi:10.1093/immadv/ltad029)
Supplement: ltad029_suppl_Supplementary_Tables_S1_Figures_S1-S4 [file ltad029_suppl_supplementary_tables_s1_figures_s1-s4.docx]

Electronic supplementary material

***Ex Vivo* Comparative Immunogenicity Assessment (EVCIA) to Determine Relative Immunogenicity in Chronic Plaque Psoriasis in Participants Receiving Humira® or Undergoing Repeated Switches Between Humira and AVT02**

Kathleen Richter^1^, Halimu N. Haliduola^1^, Jana Schockaert^2^, Aurélie Mazy^2^, Nataliya Reznichenko^3^, Eric Guenzi^1^, Fausto Berti^4^

^1^Alvotech Germany GmbH, Jülich, Germany; ^2^ImmunXperts, Gosselies, Belgium; ^3^Military Hospital (Military Unit A3309) of Military-Medical Clinical Center of Southern Region, Zaporizhzhia, Ukraine; ^4^Alvotech Swiss AG, Zürich, Switzerland

**Corresponding author**

Kathleen Richter: Kathleen.Richter@alvotech.com

**Running heading:**

Relative *Ex Vivo* Immunogenicity between Humira^®^ and AVT02

Supplementary Table S1. Overview of response to AVT02 and reference product stimulation in switching arm versus non-switching arm participants.

Equivalence limits, average fold changes, and 90% CI for equivalence are shown.

| **Stimulation** | **Time** | **Fold change** | **Lower 90% CI** | **Upper 90% CI** | **Lower equivalence limit** | **Upper equivalence limit** |
| --- | --- | --- | --- | --- | --- | --- |
| AVT02 | Week 1 | 0.99 | 0.65 | 1.50 | 0.56 | 1.77 |
| Humira^®^ | Week 1 | 1.01 | 0.68 | 1.49 | 0.45 | 2.24 |
| KLH | Week 1 | 0.94 | 0.40 | 2.22 | 0.34 | 2.97 |
| AVT02 | Week 12 | 1.06 | 0.71 | 1.59 | 0.43 | 2.33 |
| Humira^®^ | Week 12 | 1.22 | 0.77 | 1.94 | 0.31 | 3.18 |
| KLH | Week 12 | 1.86 | 0.72 | 4.78 | 0.10 | 10.21 |
| AVT02 | Week 16 | 1.34 | 0.87 | 2.07 | 0.36 | 2.74 |
| Humira^®^ | Week 16 | 1.19 | 0.86 | 1.65 | 0.45 | 2.21 |
| KLH | Week 16 | 1.38 | 0.77 | 2.51 | 0.28 | 3.54 |
| AVT02 | Week 28 | 0.65 | 0.46 | 0.94 | 0.60 | 1.66 |
| Humira^®^ | Week 28 | 0.83 | 0.64 | 1.09 | 0.66 | 1.51 |
| KLH | Week 28 | 0.67 | 0.27 | 1.67 | 0.31 | 3.24 |
| AVT02 | Overall | 0.98 | 0.80 | 1.20 | 0.67 | 1.49 |
| Humira^®^ | Overall | 1.05 | 0.89 | 1.25 | 0.61 | 1.65 |
| KLH | Overall | 1.13 | 0.71 | 1.80 | 0.19 | 5.28 |

CI confidence interval, KLH keyhole limpet hemocyanin.

**Supplementary Figure S1. Cytokine release after reference product, AVT02, or KLH *ex vivo* PBMC stimulation of participant samples collected at different time points, represented as relative secretion to negative control in %, negative control was set to 100%.**

Cytokine release, after reference product Humira or AVT02 ex vivo PBMC stimulation of subject samples collected from the AVT02-GL-302 clinical study at different timepoints, is represented as relative secretion to blank in %, blank was set to 100%. Blood samples were obtained from healthy subjects at different time points (Figure 1 – study design), PBMC’s were isolated and frozen at site, stored before sending it to the CRO (ImmunXpert). At the CRO site, cells were thawed and re-exposed to reference product Humira, AVT02, KLH, reference product Humira + KLH or AVT02 + KLH for 24h and supernatant was collected, frozen and shipped to the Alvotech Germany GmbH site.

Multiplex MSD assays were performed to determine a) IFN-γ, b) IL-6, c) IL-10, d) IL-13, e) IL-8, f) IL-1β and g) MCP-1 cytokine secretion as a relative secretion with respect to the negative control (medium). Data points represent the average of two technical replicates per sample, the median of all data points per treatment is represented as a bar. Participants assigned to the non-switching arm (dark grey bar) or switching arm (orange bar) with *n* = 20 at Baseline and Week 12, divided in *n* = 10 per treatment arm after Week 12. Re-exposure samples are represented as: dark grey dots for reference product Humira^®^ re-exposure, orange dots with grey border for AVT02 re-exposure, and white dots with red border for KLH re-exposure. Kruskal-Wallis test – multiple comparison of reference product Humira^®^ or AVT02 to positive control KLH: *p* value of values: * ≤ 0.05, ** ≤ 0.01, *** < 0.001, **** < 0.0001, ns = not significant calculated using all data points per time point. Additional statistics were applied to the comparison between reference product Humira^®^ and AVT02 re-exposure withing baseline and week 12 timepoints, and to the comparison within reference product Humira^®^ or AVT02 re-exposure of week 16 and week 28: *p* value of values: § ≤ 0.05, §§ ≤ 0.01, §§§ < 0.001, §§§§ < 0.0001.

*IFN-γ* interferon gamma*, IL* interleukin*, KLH* keyhole limpet hemocyanin, *MCP-1* monocyte chemoattractant protein-1, *MSD,* Meso Scale Discovery, *PBMC* peripheral blood mononuclear cell.

**Supplementary Figure S2. EVCIA biomarker comparison between non-switching and switching arm.**

Multiple unpaired t-test comparison: *p* value of values: * ≤ 0.05, ** ≤ 0.001, *** < 0.001, **** < 0.0001 calculated using all data points per time point. For further description see Supplementary Figure S1.


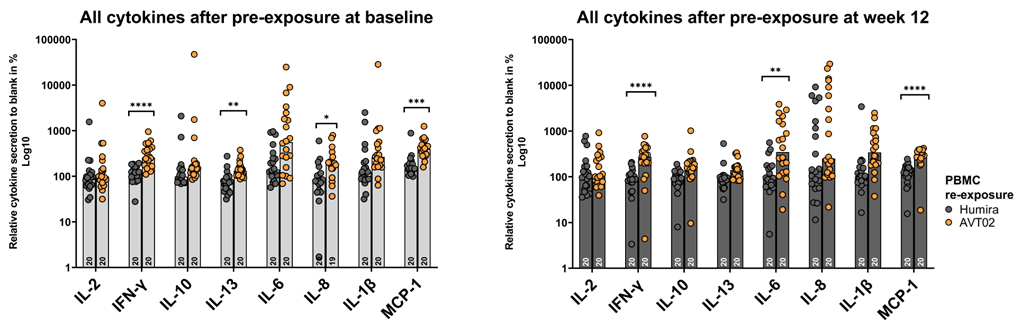


*EVCIA* ex vivo comparative immunogenicity assessment*,* *IFN-γ* interferon gamma, *IL-* interleukin*,*

*MCP-1* monocyte chemoattractant protein-1, *PBMC* peripheral blood mononuclear cell.

**Supplementary Figure S3.** **Comparison of cytokine secretion between non-switching and switching arms in participant samples collected at different timepoints after reference product, AVT02, or KLH *ex vivo* PBMC stimulation: a) IFN-γ, b) IL-6, c) IL-10, d) IL-13, e) IL-8, f) IL-1β, g) MCP-1.**

Cytokine secretion is represented as relative secretion to negative control in %, negative control was set to 100%. For further description see Supplementary Figure S1.

*IFN-γ* interferon gamma, *IL* interleukin, *KLH* keyhole limpet hemocyanin, *MCP-1* monocyte chemoattractant protein-1, *PBMC* peripheral blood mononuclear cell.

**Supplementary Figure S4.** **Comparison of cytokine secretion between non-switching and switching arms in participant samples collected at different timepoints after reference product+KLH, AVT02+KLH, or KLH *ex vivo* PBMC stimulation: a) IL-2, b) IFN-γ, c) IL-6, d) IL-10, e) IL-13, f) IL-8, g) IL-1β, h) MCP-1.**

Cytokine secretion is represented as relative secretion to negative control in %, negative control was set to 100%. For further description see Fig. S1. Kruskal-Wallis test – multiple comparison of reference product Humira®+KLH and AVT02+KLH to KLH: *p* value of values: * ≤ 0.05, ** ≤ 0.01, *** < 0.001, **** < 0.0001 calculated using all data points per time point. Additional statistics were applied to the comparison between reference product Humira^®^+KLH and AVT02+KLH re-exposure withing baseline and week 12 timepoints, and to the comparison within reference product Humira^®^+KLH or AVT02+KLH re-exposure of week 16 and week 28: *p* value of values: § ≤ 0.05, §§ ≤ 0.01, §§§ < 0.001, §§§§ < 0.0001.
